# Draft Genome Sequence of the Symbiotically Competent Cyanobacterium *Nostoc* sp. Strain KVJ20

**DOI:** 10.1128/MRA.01190-19

**Published:** 2019-11-07

**Authors:** Marie-Josée H. Halsør, Anton Liaimer, Seila Pandur, Inger L. U. Ræder, Arne O. Smalås, Bjørn Altermark

**Affiliations:** a NorStruct, Department of Chemistry, Faculty of Science and Technology, UiT The Arctic University of Norway, Tromsø, Norway; b Department of Arctic and Marine Biology, Faculty of Biosciences, Fisheries and Economics, UiT The Arctic University of Norway, Tromsø, Norway; University of Rochester School of Medicine and Dentistry

## Abstract

Nostoc sp. strain KVJ20 was isolated from the symbiotic organs of the liverwort Blasia pusilla. This cyanobacterium has been shown to have broad symbiotic competence, and bacterial extracts have inhibitory effects on cancer cell lines and microbes. An array of genes for the production of secondary metabolites is present.

## ANNOUNCEMENT

Nostoc sp. strain KVJ20 was isolated from the symbiotic organs of the liverwort Blasia pusilla L., found as a weed in a plant school on Kvaløya Island in northern Norway (1). During the isolation process, the strain was unusually clean from the first steps of cultivation and did not require additional treatments to bring it into an axenic state. This was an indication that the organism produces antibiotic compounds. We had a similar experience with the genetically similar *Nostoc* sp. strain SKSF3, which originated from soil. Metabolic profiling by matrix-assisted laser desorption ionization–time of flight mass spectrometry (MALDI-TOF MS) showed similarities between the secondary metabolite sets produced by these two isolates (1). In addition, the cell extracts of *Nostoc* sp. KVJ20 showed inhibitory effects on the A2068 metastatic human melanoma cell line and MRC5 fibroblasts (2). Insights into the metabolic capacities of the strain, based on full-genome sequencing, may help explain its antimicrobial and anticancer properties. *Nostoc* sp. KVJ20 has broad symbiotic competence and has successfully infected seedlings of the angiosperm Gunnera manicata Linden, where it existed as an intracellular symbiont (3). Thus, the genome will add valuable information on the core features underlying the symbiotic capacity of the genus.

**FIG 1:**
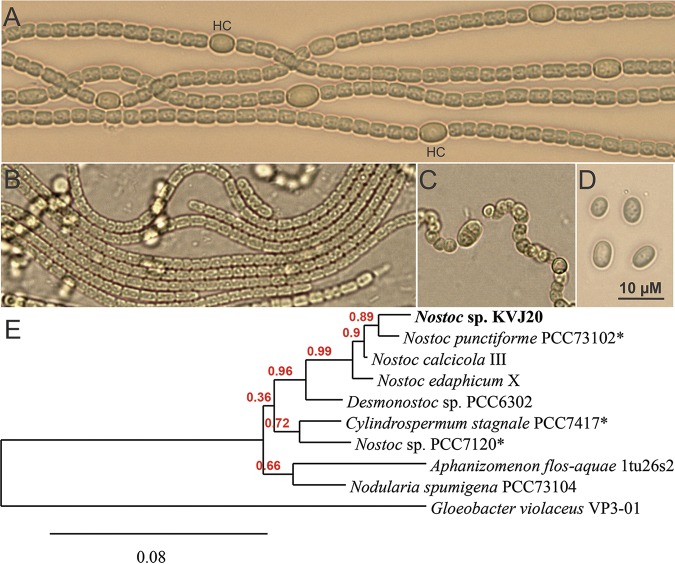
*Nostoc* sp. KVJ20 at different life stages. (A) Diazotrophically grown *Nostoc* sp. KVJ20 at exponential stage, with vegetative cells arranged in multicellular filaments interspersed by nitrogen-fixing cells and heterocysts (HC). (B) Motile filaments and hormogonia. (C and D) A filament differentiating resting cells and akinetes (C) and mature resting cells and akinetes (D). (E) Phylogenetic tree highlighting the position of *Nostoc* sp. KVJ20 (in bold) relative to other strains within the Nostocaceae family. The genomes of strain names marked with an asterisk (*) have been sequenced. The strains and their corresponding GenBank accession numbers for 16S rRNA genes are *Nostoc* sp. KVJ20, LSSA00000000; Nostoc punctiforme PCC73102, AF027655; Nostoc calcicola III, AJ630447; Nostoc edaphicum X, AJ630449; Desmonostoc sp. strain PCC6302, HG004582; Cylindrospermum stagnale PCC7417, AJ133163; *Nostoc* sp. strain PCC7120, BA000019; Aphanizomenon flos-aquae 1tu26s2, AJ630443; and Nodularia spumigena PCC73104, DQ185241. 16S rRNA genes, aligned using MUSCLE (10, 11), were used as input to the Web server Phylogeny.fr (12). A total of 1,402 positions, not including gaps, were compared and used to reconstruct and analyze the phylogenetic relationships. Confidence index values are shown in red, and the bar represents the amount of nucleotide substitutions per site. Gloeobacter violaceus (GenBank accession number FR798924) was used as an outgroup.

Isolation and culturing of *Nostoc* sp. KVJ20 from the symbiotic organs of the liverwort *Blasia pusilla* were carried out as described earlier (1, 4). The strain is maintained at the Department of Arctic and Marine Biology, Faculty of Biosciences, Fisheries and Economics, UiT The Arctic University of Norway. Photos of *Nostoc* sp. KVJ20 at different life stages and the phylogenetic placement are presented in Fig. 1.

Genomic DNA from *Nostoc* sp. KVJ20 was purified according to the method for bacterial genomic DNA isolation using CTAB, v3 (5). The only modification is two washes in 5 M NaCl preceding the DNA isolation. The DNA was quantified using a Qubit fluorometer. For the library, a Nextera DNA library prep kit (catalog number FC-121-1031) was used. The library was sequenced on a MiSeq machine (Illumina) at UiT The Artic University of Norway using the MiSeq reagent kit v3 (2 × 300 bp; catalog number MS-102-3003). The reads were quality trimmed, adapter trimmed, and assembled using CLC Genomics Workbench 8.5 (Qiagen) with default settings. A total of 9,259,128 reads with an average read length of 208 bases were assembled. The NCBI Prokaryotic Genome Annotation Pipeline v3.1 was used for annotation, with GeneMarkS+ used for gene identification (6, 7). The draft genome is composed of 425 contigs (332 scaffolds), with an *N*_50_ value of 41,001 bp, a total size of 9.2 Mbp, an average read coverage of 200×, and a GC content of 41.69%. In total, 7,676 genes were predicted, with 7,210 coding sequences (CDSs), 104 RNAs, and 362 pseudogenes.

Analysis using the antiSMASH tool v4.0 (8), complemented by BLAST (9), using default settings, predicted 19 gene clusters containing genes involved in the biosynthesis of nonribosomal peptides, polyketides, and ribosomally synthesized and posttranslationally modified peptides.

The symbiotic competence, production of antibacterial and anticancer compounds, cellular differentiation mechanism, and how these properties are connected to the production of secondary metabolites from the identified gene clusters will be interesting to pursue further.

### Data availability.

The genome sequence is deposited in the NCBI under BioProject number PRJNA310825, SRA project number SRS4677145, and BioSample number SAMN04453661. The assembled genome is deposited under GenBank accession number LSSA00000000.

## References

[1] Liaimer A, Jensen JB, Dittmann E 2016 A genetic and chemical perspective on symbiotic recruitment of cyanobacteria of the genus *Nostoc* into the host plant *Blasia pusilla* L. Front Microbiol 7:1693. doi:10.3389/fmicb.2016.01693. 27847500 PMC5088731

[2] Liaimer A, Helfrich EJ, Hinrichs K, Guljamow A, Ishida K, Hertweck C, Dittmann E 2015 Nostopeptolide plays a governing role during cellular differentiation of the symbiotic cyanobacterium *Nostoc punctiforme*. Proc Natl Acad Sci U S A 112:1862–1867. doi:10.1073/pnas.1419543112. 25624477 PMC4330735

[3] Warshan D, Liaimer A, Pederson E, Kim SY, Shapiro N, Woyke T, Altermark B, Pawlowski K, Weyman PD, Dupont CL, Rasmussen U 2018 Genomic changes associated with the evolutionary transitions of Nostoc to a plant symbiont. Mol Biol Evol 35:1160–1175. doi:10.1093/molbev/msy029. 29554291 PMC5913679

[4] West NJ, Adams DG 1997 Phenotypic and genotypic comparison of symbiotic and free-living cyanobacteria from a single field site. Appl Environ Microbiol 63:4479–4484. 16535734 10.1128/aem.63.11.4479-4484.1997 PMC1389290

[5] Joint Genome Institute. 2012 Bacterial genomic DNA isolation using CTAB. DOE Joint Genome Institute, Walnut Park, CA http://1ofdmq2n8tc36m6i46scovo2e-wpengine.netdna-ssl.com/wp-content/uploads/2014/02/JGI-Bacterial-DNA-isolation-CTAB-Protocol-2012.pdf.

[6] Tatusova T, DiCuccio M, Badretdin A, Chetvernin V, Nawrocki EP, Zaslavsky L, Lomsadze A, Pruitt KD, Borodovsky M, Ostell J 2016 NCBI Prokaryotic Genome Annotation Pipeline. Nucleic Acids Res 44:6614–6624. doi:10.1093/nar/gkw569. 27342282 PMC5001611

[7] Borodovsky M, Lomsadze A 2011 Gene identification in prokaryotic genomes, phages, metagenomes, and EST sequences with GeneMarkS suite. Curr Protoc Microbiol Chapter 4:Unit 4.5.1-17. doi:10.1002/0471250953.bi0405s35. 24510847

[8] Blin K, Wolf T, Chevrette MG, Lu X, Schwalen CJ, Kautsar SA, Suarez Duran HG, de Los Santos ELC, Kim HU, Nave M, Dickschat JS, Mitchell DA, Shelest E, Breitling R, Takano E, Lee SY, Weber T, Medema MH 2017 antiSMASH 4.0—improvements in chemistry prediction and gene cluster boundary identification. Nucleic Acids Res 45:W36–W41. doi:10.1093/nar/gkx319. 28460038 PMC5570095

[9] Altschul SF, Gish W, Miller W, Myers EW, Lipman DJ 1990 Basic local alignment search tool. J Mol Biol 215:403–410. doi:10.1016/S0022-2836(05)80360-2. 2231712

[10] Edgar RC 2004 MUSCLE: a multiple sequence alignment method with reduced time and space complexity. BMC Bioinformatics 5:113. doi:10.1186/1471-2105-5-113. 15318951 PMC517706

[11] Edgar RC 2004 MUSCLE: multiple sequence alignment with high accuracy and high throughput. Nucleic Acids Res 32:1792–1797. doi:10.1093/nar/gkh340. 15034147 PMC390337

[12] Dereeper A, Guignon V, Blanc G, Audic S, Buffet S, Chevenet F, Dufayard JF, Guindon S, Lefort V, Lescot M, Claverie JM, Gascuel O 2008 Phylogeny.fr: robust phylogenetic analysis for the non-specialist. Nucleic Acids Res 36:W465–W469. doi:10.1093/nar/gkn180. 18424797 PMC2447785

